# Supplementary material for: Dysbiosis by Eradication of Helicobacter pylori Infection Associated with Follicular Gastropathy and Pangastropathy
Source: Microorganisms. 2023 Nov 10;11(11):2748. doi: 10.3390/microorganisms11112748 (PMC10673246; doi:10.3390/microorganisms11112748)
Supplement: Supplementary file 1 [file microorganisms-11-02748-s001.zip › Supplementary Figure S3.pdf]

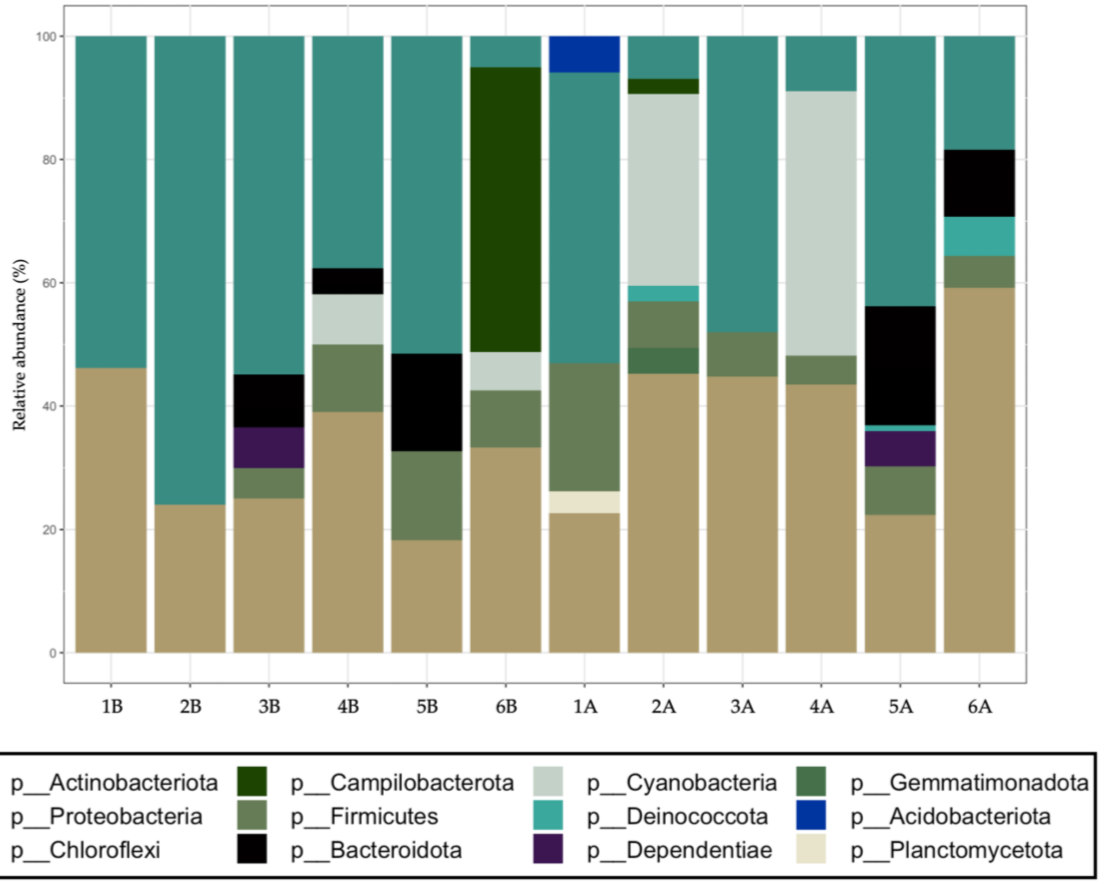

Supplementary Figure S3. Metagenomic profile at Phylum level of patients diagnosed with follicular gastropathy and pangastropathy initially associated to infection with *H. pylori*. B: Pre-treatment patient; **A**: Posttreatment patient.
