# Supplementary material for: Dysbiosis by Eradication of Helicobacter pylori Infection Associated with Follicular Gastropathy and Pangastropathy
Source: Microorganisms. 2023 Nov 10;11(11):2748. doi: 10.3390/microorganisms11112748 (PMC10673246; doi:10.3390/microorganisms11112748)
Supplement: Supplementary file 1 [file microorganisms-11-02748-s001.zip › Supplementary Figure S4.pdf]

A

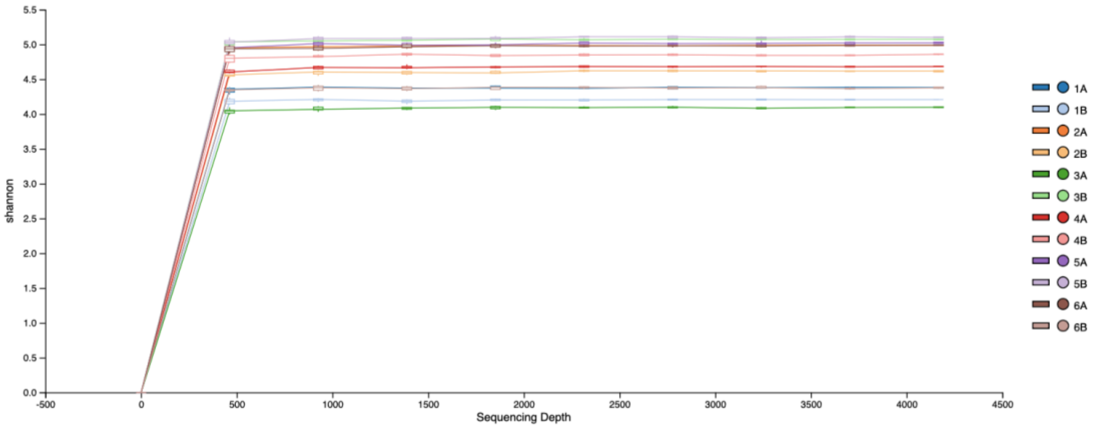

B

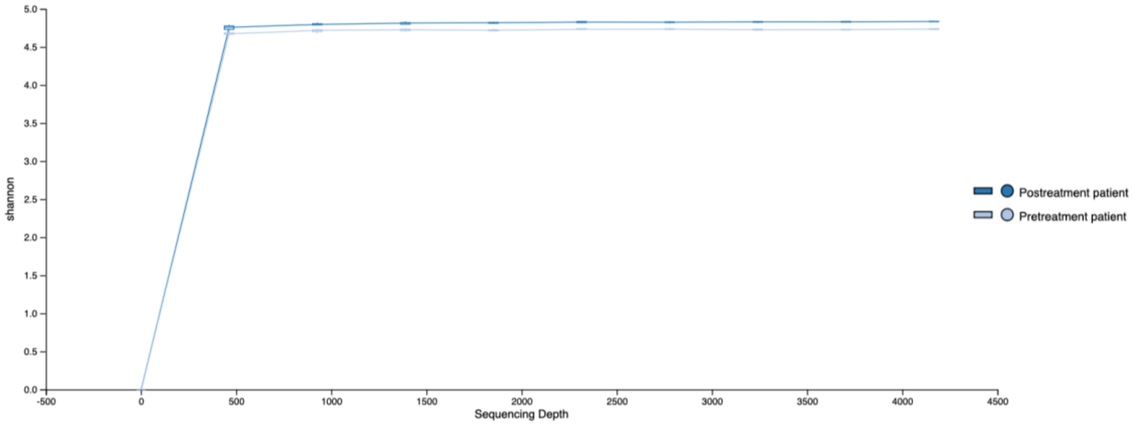

Supplementary Figure S4. Alpha rarefaction for Shannon's H index. A: Rarefaction curve by patient (B: Pre-treatment patient; A: Posttreatment patient). **B**: Rarefaction curve by treatment status
