# Supplementary material for: Dysbiosis by Eradication of Helicobacter pylori Infection Associated with Follicular Gastropathy and Pangastropathy
Source: Microorganisms. 2023 Nov 10;11(11):2748. doi: 10.3390/microorganisms11112748 (PMC10673246; doi:10.3390/microorganisms11112748)
Supplement: Supplementary file 1 [file microorganisms-11-02748-s001.zip › Supplementary Table S1.pdf]

Supplementary Table S1. Histological findings of the FFPE gastric biopsies for evaluation of the presence of *C. acnes* and *H. pylori*.

Patient	Clinical diagnosis
**1**	Gastritis
**2**	Mild chronic gastritis in gastric fundus and corpus.<br>Nodular gastritis in gastric antrum.
**3**	Inactive follicular gastritis. No atrophy
**4**	Gastritis
**5**	Mild gastritis
**6**	Mild chronic gastritis. No atrophy.
**7**	Reactive gastritis in gastric antrum. Billiard reflux.
**8**	Gastric fundus and antrum with no significative alterations.
**9**	Mild chronic gastritis. No atrophy.
**10**	Superficial gastritis in gastric fundus and corpus. Mild gastritis.<br>Mild atrophy in gastric antrum.
**11**	Superficial gastritis. No atrophy.
**12**	Minimal gastritis in gastric antrum.
**13**	Gastritis in gastric antrum. Moderate activity<br>Superficial gastritis in gastric corpus. No activity.
**14**	Gastritis in gastric antrum. Moderate activity. No atrophy.
**15**	Chronic gastritis associated to *H. pylori* infection. Moderate activity.
**16**	Superficial chronic gastritis in gastric fundus, corpus and antrum. No activity.
**17**	Gastritis in gastric antrum. Mild atrophy.
**18**	Mild gastritis in gastric fundus and corpus. No activity.<br>Follicular active gastritis in gastric antrum, associated to *H. pylori* infection.
**19**	Minimal histologic alterations in gastric fundus, corpus and antrum.
**20**	Esophagitis
**21**	Minimal histologic alterations in gastric antrum.
**22**	Enteropathy
**23**	Chronic follicular gastritis associated to *H. pylori* infection. No activity.
**24**	Follicular gastropathy in gastric corpus associated to *H. pylori* infection.
**25**	Follicular pangastropathy associated to *H. pylori* infection.
**26**	Follicular gastropathy in gastric corpus associated to *H. pylori* infection.
**27**	Follicular gastropathy in gastric corpus associated to *H. pylori* infection.
**28**	Follicular pangastropathy associated to *H. pylori* infection.
**29**	Follicular pangastropathy associated to *H. pylori* infection.
**30 – 40**	Intestinal metaplasia
