# Supplementary material for: Dysbiosis by Eradication of Helicobacter pylori Infection Associated with Follicular Gastropathy and Pangastropathy
Source: Microorganisms. 2023 Nov 10;11(11):2748. doi: 10.3390/microorganisms11112748 (PMC10673246; doi:10.3390/microorganisms11112748)
Supplement: Supplementary file 1 [file microorganisms-11-02748-s001.zip › Supplementary Table S2.pdf]

Supplementary Table S2. Evaluation of susceptibility to four different antibiotics in three primary cultures of *H. pylori* isolated from three *H. pylori* patients with follicular gastropathy and pangastropathy.

Antibiotic	MIC (μg/mL)		
	**1B**	**5B**	**6B**
**CLR**	0.03125	0.25	> 64
**AMX**	> 64	0.25	32
**LVX**	> 64	0.5	8
**MTZ**	> 1024	64	128

B: Pre-treatment patient.

CLR: Clarithromycin; AMX: Amoxicillin; LVX: Levofloxacin; MTZ: Metronidazole.
