# Supplementary material for: Dysbiosis by Eradication of Helicobacter pylori Infection Associated with Follicular Gastropathy and Pangastropathy
Source: Microorganisms. 2023 Nov 10;11(11):2748. doi: 10.3390/microorganisms11112748 (PMC10673246; doi:10.3390/microorganisms11112748)
Supplement: Supplementary file 1 [file microorganisms-11-02748-s001.zip › Supplementary Table S3.pdf]

Supplementary Table S3. Frequency of resistance in the *H. pylori* subcultures.

Antibiotic	Interpretative categories	MIC breakpoints (µg/mL)	Frequency (%)
**CLR**	S	≤ 0.25	40
	I	0.5	7.27
	R	≥ 1	52.72
**AMX**	S	< 4	69.09
	I	-	-
	R	≥ 4	30.91
**LVX**	S	< 1	38.18
	I	-	-
	R	≥ 1	61.82
**MTZ**	S	< 2	1.81
	I	2 – 4	10.91
	Low resistance	> 8	12.72
	Moderate resistance	32 – 64	21.82
	High resistance	128	52.73

CLR: Clarithromycin; AMX: Amoxicillin; LVX: Levofloxacin; MTZ: Metronidazole.
S: Sensitive; I: Intermediate; R: Resistant.
