# Supplementary material for: Dysbiosis by Eradication of Helicobacter pylori Infection Associated with Follicular Gastropathy and Pangastropathy
Source: Microorganisms. 2023 Nov 10;11(11):2748. doi: 10.3390/microorganisms11112748 (PMC10673246; doi:10.3390/microorganisms11112748)
Supplement: Supplementary file 1 [file microorganisms-11-02748-s001.zip › Supplementary Table S4.pdf]

Supplementary Table S4. Frequency of genotypes in *H. pylori* strains isolated from the study subjects before the administration of STT.

Patient	Gene		Frequency (%)	Genotype	Frequency (%)
1B	*cagA+*		81	*cagA+ vacAs1+m1+m2+**	54
	vacA	*s1+*	100	*cagA+ vacAs1+m1–m2–*	9
		m1+	27	*cagA+ vacAs1+m1+**	18
		m1+m2+	54	*cagA– vacAs1+m1–m2–*	9
		m1-m2-	18	*cagA– vacAs1+m1+**	9
5B	*cagA+*		90	*cagA+ vacAs1+m1+m2+*	50
	vacA	*s1+*	70		
		s2+	30	*cagA+ vacAs1+m2+*	20
		m1+	10	*cagA+ vacAs2+m1+m2+**	10
		m2+	30	*cagA+ vacAs2+m1+*	10
		m1+m2+	60	*cagA– vacAs2+m2+*	10
6B	*cagA+*		66	*cagA+ vacAs1+m1+*	58
	vacA	*s1+*	92	*cagA+ vacAs2+m1+m2*	8
		m1+	66	*cagA– vacAs1+m1+m2+*	26
		m1+m2+	34	*cagA– vacAs1+m1+*	8

B: Pre-treatment patient.

*: Genotypes observed in more than one patient.

cagA: cytotoxin CagA gene; *vacA*: vacuolating cytotoxin VacA gene.

s: signal-sequence region (*s*-region); *m*: mid-region (*m*-region).
